# Hematological Parameters Among Adult Patients Diagnosed With Type 2 Diabetes Mellitus at Jimma University Medical Center, Jimma, Southwest, Ethiopia

**DOI:** 10.1155/ah/2225431

**Published:** 2025-08-04

**Authors:** Edosa Tadasa, Eman Kemal

**Affiliations:** School of Medical Laboratory Sciences, Faculty of Health Sciences, Jimma University, Jimma, Oromia, Ethiopia

**Keywords:** hematological parameters, JUMC, type 2 diabetes mellitus

## Abstract

**Background:** Diabetes is a significant worldwide health challenge associated with significant metabolic, cellular, and hematological disturbances. Hematological alterations are well-documented complications of diabetes and play a crucial role in the progression of diabetes related pathology. While extensive data exist globally on hematological parameters in type 2 diabetes mellitus (T2DM), specific insights into these parameters and their local determinants within study area remain limited. Therefore, this study aimed to assess the hematological parameters among adult patients diagnosed with T2DM in JUMC at Jimma, Southwest, Ethiopia, 2024.

**Methods:** A total of 200 medical charts of adults with T2DM who registered for follow-up at Jimma University Medical Center were reviewed from December 2023 to February 2024. Data were collected using a data extraction checklist. Bivariate and multivariate logistic regression analyses were performed to identify factors associated with hematologic abnormalities. A *p* value less than 0.05 indicates statistical significance.

**Result:** The overall prevalence of anemia and leukocytosis in adults with T2DM was 14.0% and 12.0%, respectively. Neutrophilia was the common white blood cell (WBC) abnormality detected in 9.5% of the patients. Besides, thrombocytopenia and thrombocytosis were observed in 2.5% and 1.5% of the patients, respectively. Increasing age 5.28 (95% CI: 1.07–26.1) and duration of diabetes mellitus (≥ 3 years) (AOD = 3.1 (95% CI: 1.02–9.5)) were significantly associated with anemia and leukocytosis, respectively.

**Conclusion:** This study found a prevalence of hematological abnormalities in adults with T2DM, including anemia, elevated WBC count, increased neutrophils, and thrombocytopenia. Anemia was associated with advanced age, while leukocytosis was associated with a longer diabetes duration. Therefore, it is recommended to start regularly screening T2DM patients for hematological abnormalities to improve clinical practice, guide treatment decisions, and develop targeted interventions.

## 1. Introduction

Diabetes mellitus (DM) is a chronic condition characterized by elevated blood glucose levels due to insufficient production or utilization of insulin in the body [1]. It encompasses a diverse range of metabolic disorders that impact the regulation of carbohydrate, fat, and protein metabolism [2]. Poorly managed hyperglycemia can lead to severe complications affecting various organs, including cardiovascular diseases (CVD), nerve damage, kidney issues, lower limb amputations, and eye problems that can result in vision loss and blindness [1, 2].

Diabetes is classified into two main types: type 1 diabetes mellitus (T1DM) and type 2 diabetes mellitus (T2DM) [3]. T1DM occurs when the immune system attacks the pancreas' beta cells, leading to a lack of insulin production. The exact causes are not fully understood but involve genetic susceptibility and environmental triggers [4]. T2DM is responsible for the majority of diabetes cases and is prevalent in low- and middle-income countries. It is primarily caused by insulin resistance, where cells do not respond properly to hormone insulin, leading to high blood sugar levels. Various factors, such as genetics, excess body fat, inactivity, and poor diet, can contribute to insulin resistance and it primarily affects adults [3].

Patients with T2DM have a higher risk of CVD due to atherogenic dyslipidemia, coronary artery disease, and myocardial infarction [5, 6]. High blood sugar levels in DM cause disruptions in cellular metabolism, resulting in increased production of reactive oxygen species (ROS) and nonenzymatic glycation of macromolecules. These disturbances alter cellular structure and function and lead to the formation of advanced glycation end products. Advanced glycation end products worsen metabolic disturbances and increase ROS production by interacting with specific receptors. These changes affect the basement membrane, leading to modifications in blood vessel permeability and vasodilation [7–9].

Hematological parameters encompass measurements and characteristics of blood components, such as red blood cells (RBC), white blood cells (WBCs), and platelets (PLT). They include RBC count, hemoglobin level (Hgb), hematocrit (Hct), WBC count, PLT count, and related indices. These parameters serve as crucial indicators of overall health, offering valuable insights into various diseases and conditions, including diabetes and its complications [10].

Alterations in blood characteristics in diabetes can be caused by various factors, such as elevated ROS levels which lead to oxidative stress, which is associated with tissue damage and changes in blood components such as RBCs, PLTs, and endothelial cells. These changes can result in complications such as anemia and increased blood clotting tendency and contribute to heart disease in individuals with diabetes [10–12]. Insulin resistance is another factor linked to endothelial dysfunction, heightened inflammation markers, and increased PLT activity, all of which can hasten vascular issues in T2DM patients [11]. Recent attention has been on blood parameters like WBC count, RBC distribution width (RDW), mean platelet volume (MPV), platelet distribution width (PDW), and PLT count as markers of endothelial dysfunction and inflammation in T2DM [13, 14]. An elevated WBC count is a classic sign of inflammation, with research indicating a relation between WBC count and diabetes risk [15]. PLTs play a crucial role in maintaining normal blood balance, with MPV serving as an indicator of their function [16]. Complications related to diabetes, such as accelerated artery hardening, involve PLT activation, which contributes to inflammation and the development of CVD in T2DM patients [17].

T2DM is a chronic metabolic disorder characterized by insulin resistance and high blood sugar levels. While the impact of diabetes on various organ systems has been extensively studied, there is a need to explore the specific hematological changes that occur in patients with T2DM [18]. According to the Global Health Estimates 2016, DM has been considered as one of the leading 10 causes of death in low-, middle-, and upper-income countries [19].

Hematological abnormalities, such as decreased or increased RBCs and Hgb levels, can serve as indicators for anemia or polycythemia, respectively. Anemia is characterized by low RBC count or Hgb levels and is more prevalent with T2DM [20]. In older patients, there is a positive association between packed cell volume (PCV), Hgb, RBC indices, and the presence of anemia. Other parameters, such as mean cell volume (MCV), mean cell hemoglobin (MCH), and mean cell hemoglobin concentration (MCHC), are calculated mathematically to provide information about the concentration of Hgb and the size of RBCs. Diabetes often impairs the body's immune system. Leukocytosis has been found to be correlated with the severity of infection, while reduced WBC (leukopenia) is associated with inflammatory lesions. PLT indices play a role in clotting and fibrinolytic activity, playing an important role in diabetic complications such as atherosclerosis and vascular disease. In addition, these PLT indices are useful biomarkers in the early diagnosis and prognosis of T2DM [19, 21].

Hematological changes are frequently seen in individuals with T2DM. However, current guidelines for managing diabetes do not recommend routine monitoring of hematological parameters. Research conducted in various countries has yielded conflicting findings regarding the hematological profiles of diabetic individuals. Some studies have shown no notable differences in RBC indices, WBC count, and PLT count between diabetic patients and healthy controls. Conversely, other studies have identified significantly elevated RBC, WBC, and PLT indices in diabetic patients compared with nondiabetic individuals. In addition, there are studies indicating significantly lower RBC indices (excluding RDW) and higher WBC and PLT indices in diabetic patients when compared with controls [22].

Hematological parameters are not commonly used as routine diagnostic biomarkers for monitoring diabetes and its complications in developing countries like Ethiopia. Therefore, conducting a retrospective study on hematological parameters among adult patients with T2DM in these contexts would help fill knowledge gaps and provide valuable insights into the hematological profile of diabetic patients in these regions. The hematological parameters have the potential to serve as a cost-effective and readily available method for monitoring and predicting health outcomes in patients with T2DM. To gain a better understanding of this relationship, this study aimed to assess the hematological profile of individuals with T2DM. Detecting and treating these issues, like anemia, promptly can improve health outcomes and quality of life for diabetic individuals [22].

## 2. Materials and Methods

### 2.1. Study Area, Design, and Period

This study was conducted at Jimma University Medical Center (JUMC) in Jimma City, which is about 352 km southwest of Addis Ababa, the capital of Ethiopia. It serves as the sole teaching and medical hospital in the southwestern region, providing healthcare services to a large population. It gives service to more than 1000 DM patients. A retrospective cross-sectional study was conducted from December 2023 to February 2024.

### 2.2. Study Participants

All patients with T2DM who had been registered in the DM clinic of JUMC were used as the Source population. The study population included individuals with T2DM who had a registration list in their medical records at the JUMC, containing follow-up dates at the time of data collection, and who met the specified inclusion criteria.

### 2.3. Eligibility Criteria

The study included all medical records of patients with T2DM above the age of 18 for at least 3 months prior to the data collection and who had regular follow-ups. Meanwhile, T2DM patients with known hematological disorders such as anemia, leukemia, acute or chronic infections, thyroid abnormalities, hypertension, and autoimmune disorders, as well as those who had received blood in the last 3 months, and pregnant women, were excluded from the study.

### 2.4. Sample Size and Sampling Technique

The sample size calculation was not applicable because the study used a retrospective cross-sectional design. This study included 200 adults with T2DM who had at least 3 months of follow-up data prior to the data collection period and had complete data on key variables.

### 2.5. Data Collection Techniques and Tools

Data collection was carried out using a structured data extraction checklist. Data sources included patient admission forms, follow-up cards, and DM registration books. The data extraction sheet consisted of sex, age, BMI, duration of DM, FBS level, type of medication, and hematological parameters. Hematological parameters, including RBC, Hgb, Hct, MCH, MCHC, MCV, RDW, WBC, WBC differential count, PLT count, MPV, PCT, and PDW were collected.

### 2.6. Data Quality Assurance

The accuracy and completeness of the extracted data were verified by conducting quality checks and cross-referencing with patient records. Any inconsistencies or missing data were addressed through appropriate measures, like contacting the healthcare providers if necessary.

### 2.7. Data Analysis and Interpretation

The data were analyzed using the Statistical Package for Social Science (SPSS) Version 20 software. Bivariate logistic regression was conducted to assess the strength of the association between the dependent and independent variables. Variables with a value of < 0.25 in the bivariate analysis were fitted into the multivariate logistic regression to adjust for the confounding factors. A significance level of *p* < 0.05 was used to determine statistical significance.

### 2.8. Operational Definitions

Anemia: Hgb values < 12 g/dL for females and < 13 g/dL for males [23].

Leukocytosis: a total WBC count > 10,000 cells/μL. Leukopenia: a total WBC count < 4000 cells/μL. Lymphocytosis: a lymphocyte count > 4500 cells/μL. Lymphopenia: a total lymphocyte count < 1000 cells/μL. Neutropenia: a total neutrophil count < 1500 cells/μL. Neutrophilia: a total neutrophil count > 7000 cell/μL [24, 25].

Thrombocytosis: a total PLT count > 450,000 cells/μL. Thrombocytopenia: a total PLT count < 150,000 cells/μL [26].

## 3. Result

### 3.1. Sociodemographic and Clinical Characteristics

A total of 200 medical records of individuals diagnosed with T2DM were reviewed. The mean age of the patients was (51.87 ± 13.06) years, and most belonged to the age group over 46. More than half of the participants (116; 58.0%) were male. Around 110 (55%) of the participants resided in urban areas. The average FBS level was (172.4 ± 83.26) mg/dL, and 19 (9.5%) patients experienced hypoglycemia. T2DM patients had an average systolic blood pressure of (128.61 ± 15.75) mmHg and an average diastolic blood pressure of (86.10 ± 3.24) mmHg. Approximately 103 (51.5%) patients had been living with T2DM for three years or more since their diagnosis (Table 1).

### 3.2. Laboratory Findings

Laboratory results indicate that the Hgb levels for patients with T2DM range from 9.8 to 19.3 g/dL, with an average of 14.4 ± 1.77 g/dL. The interquartile range of WBC count was 8.25 (3.49–23.66) × 10^3^ cells/μL, ranging from 3.49 to 23.66 × 10^3^ cells/μL. In addition, PLT counts ranged from 43 to 638 × 10^3^ cells/μL, with a mean of 268.40 ± 80.92 × 10^3^ cells/μL (Table 2).

### 3.3. Magnitude of Hematological Abnormalities

**Figure 1:**
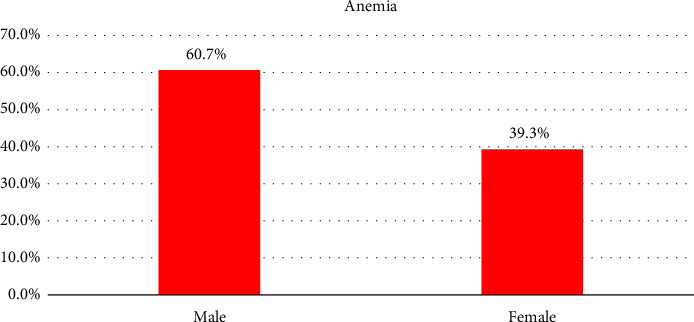
The distribution of anemia by sex among adults with T2DM at Jimma University Medical Center.

**Figure 2:**
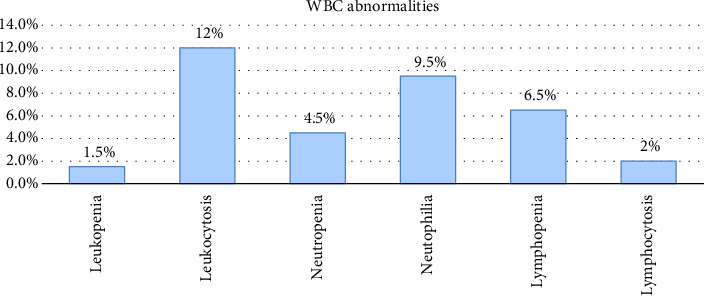
White blood cell abnormalities among adults with T2DM at Jimma University Medical Center.

The prevalence of hematological abnormalities is as follows: anemia among adults with T2DM was 14.0% with 17 (60.7%) of them being male patients (Figure 1). Thrombocytosis was found in 1.5% of the patients, while thrombocytopenia was observed in 2.5%. The other hematological abnormalities in adults with T2DM included leukocytosis, which was present in 12.0% of the patients. In addition, neutrophilia and lymphocytosis were detected in 9.5% and 2.0% of the patients, respectively (Figure 2).

### 3.4. Factors Associated With Anemia

In this study, a binary logistic regression model was used to investigate factors associated with anemia in adults with T2DM. Initially, age, types of medication, and FBS levels were analyzed by bivariate analysis, and multivariate analysis confirmed advanced age 5.28 (95% CI: 1.07–26.1) as a significant factor associated with anemia in T2DM patients (Table 3).

### 3.5. Factors Associated With Leukocytosis

To investigate the factors associated with elevated WBC counts in individuals with T2DM, both bivariate and multivariate logistic regression analyses were conducted. The analysis revealed that only a duration of diabetes ≥ 3 years (AOR = 3.1 (95% CI: 1.02–9.5)) was significantly associated with leukocytosis (Table 4).

## 4. Discussion

According to the findings of this study, it was observed that the prevalence of anemia among individuals diagnosed with T2DM was determined to be 14.0%. These data suggest that anemia can be classified as a mild public health concern among adults living with T2DM, based on the WHO's classification for public health significance of anemia ranges from 5.0% to 19.9% [23]. The prevalence of anemia observed in this study appears to be higher than the rate reported by a cross-sectional study conducted at Gonder Comprehensive Specialized Hospital, which stood at 8.02% [27] but lower than the findings from a study conducted in Nigeria, where the prevalence was reported to be 45.2% [28].

These variations in the prevalence of anemia among individuals with T2DM may be attributed to differences in the criteria used for diagnosing anemia, sample sizes, and the unique characteristics of the study population that could influence the occurrence of anemia in patients with T2DM. In our study, the study subjects were adults, and most of them were males. The etiology of anemia in T2DM is multifactorial and consists of chronic hyperglycemia, inflammation, oxidative stress, AGEs, nutritional deficiencies, drugs, and hormonal changes in addition to kidney disease [29, 30].

In this study, another hematological abnormality observed was leukocytosis, which was identified in 12.0% of the patients. Research has suggested that leukocytosis is a common occurrence in individuals with T2DM. Studies conducted in India [20], at Datta Megha College and Shalini Tai Meghe Hospital [31], as well as in Libya [32], Gonder [33], and Debre Berhan [22], have shown that the average WBC count is higher in patients with diabetes compared with nondiabetic individuals. The elevated WBC count primarily reflects increased levels of neutrophils in these studies. The underlying reason for the elevated WBC count may be linked to the heightened oxidative stress resulting from elevated levels of hyperglycemia in individuals with diabetes [1]. In addition, there is evidence suggesting that neutrophils serve as indicators of inflammation, playing a role in the advancement of diabetic complications [22].

Approximately 2.5% of the patients exhibit thrombocytopenia, characterized by a low PLT count, while conflicting data from studies conducted by Gonder [33] and Debre Berhan [22] have shown a notable rise in PLT count among individuals with T2DM. These discrepancies underscore the intricate nature and potential diversity in PLT counts within T2DM populations, emphasizing the necessity for additional research to uncover the underlying mechanisms and consequences of these variations [34].

Regarding factors associated with different hematological abnormalities, advanced age 5.28 (95% 1.07–26.1) is statistically significant with anemia. Research in Ethiopia has shown that individuals of older ages with diabetes are at a significantly higher risk of developing anemia compared with their younger counterparts [35]. This phenomenon is supported by a body of evidence from a study conducted in Malaysia [36]. The increased prevalence of anemia in older individuals with diabetes can be attributed to a combination of factors. First, as people age, there is a natural increase in the turnover of RBCs, which can lead to a higher demand for new RBC production. However, the compensatory mechanisms that regulate this process become less effective with age, potentially resulting in the development of anemia.

Moreover, the presence of DM further complicates the situation. In individuals with diabetes, the intricate balance of RBC production and destruction may be disrupted, contributing to the onset of anemia [37]. In addition, aging is associated with a decline in the secretion of erythropoietin, a hormone crucial for stimulating RBC production in the bone marrow. This decrease in erythropoietin levels can further increase the risk of anemia in older individuals with diabetes [38]. Furthermore, deficiencies in essential vitamins such as folate and cyanocobalamin, bone marrow disorders, and a higher burden of comorbidities commonly seen in older diabetic patients can also play a role in the development of anemia [39].

In this study, having diabetes for three or more years was more likely associated with leukocytosis, with an adjusted odds ratio of AOR = 3.1 (95% CI: 1.02–9.5). In a study conducted in Gondar prior to this research, it was discovered that there exists a correlation between the extended duration of diabetes and an elevated WBC count [40]. The findings indicated that diabetic patients with a prolonged history of the disease often exhibit clinically elevated levels of WBCs, which could potentially be linked to their increased vulnerability to infections [41]. This suggests that the duration of diabetes may play a crucial role in influencing the immune response and susceptibility to infections in individuals with DM [40].

This study has limitations due to its reliance on secondary data extracted from medical records. The absence of crucial predictor variables, such as patient behavior, could have influenced the outcomes. Furthermore, the cross-sectional design of the study restricts the ability to definitively establish causal relationships between variables, and there was no longitudinal follow-up, so trends or improvements/worsening with treatment or disease duration are not captured. Therefore, it is imperative to exercise caution when interpreting the findings and to account for additional factors in future research endeavors.

## 5. Conclusion

This study reveals that the prevalence of anemia was found to be 14.0%, categorizing it as a mild public health problem among adults with T2DM. Furthermore, leukocytosis was observed in 12% of the patients. Other notable abnormalities included thrombocytopenia and thrombocytosis, affecting 2.5% and 1.5% of the patients, respectively. Moreover, the study revealed that anemia was more commonly associated with advanced age in individuals with T2DM. In addition, the study found that leukocytosis was more likely to be associated with a longer duration of diabetes in these patients. Therefore, implementing regular hematological profile assessments should be a key consideration for the proper management of T2DM patients, serving as a vital step in preventing any further complications due to hematological abnormalities.

## Figures and Tables

**Table 1:** Sociodemographic and clinical characteristics of T2DM patients attending Jimma University Medical Center in Jimma, Southwest, Ethiopia, 2024.

Variables	Category	Frequency	Percentage (%)
Sex	Male	116	58
	Female	84	42

Age	18–25	8	4.0
	26–35	16	8.0
	36–45	54	27.0
	≥ 46	122	61.0

Residence	Urban	110	55
	Rural	90	45

Duration of DM diagnosis	< 3 years	97	48.5
	≥ 3 years	103	51.5

BMI	Normal	120	60.0
	Abnormal	80	40.0

FBS level	< 70 mg/dL	19	9.5
	70–125 mg/dL	73	36.5
	≥ 126 mg/dL	108	54

Type of medication	Metformin	106	53.0
	Metformin + sulfonylurea	94	47

**Table 2:** Hematological parameters of adults with T2DM patients attending Jimma University Medical Center in Jimma, Southwest, Ethiopia, 2024.

Parameter	Range	Mean ± SD	Median (IQR)
RBC (× 10^6^/μL)	3.57–6.25	4.94 ± 0.52	
Hgb (g/dL)	9.8–19.3	14.4 ± 1.77	
Hct (%)	31.1–52.3	42.5 ± 4.45	
MCV (fL)	63.0–102.0	87.32 ± 5.93	
MCH (pg)	19.8–39.3	29.75 ± 2.32	
MCHC (g/dL)	24.1–42.5	33.97 ± 2.01	
RDW (%)	7.0–62.6	42.3 ± 9.41	
WBC (× 10^3^/μL)	3.49–23.66		8.25 (3.49–23.66)
Neutrophil (× 10^3^/μL)	0.70–21.40	5.31 ± 3.36	
Lymphocyte (× 10^3^/μL)	0.04–8.61	2.31 ± 1.17	
Platelet (× 10^3^/μL)	43–638	268.40 ± 80.92	
MPV (fL)	7.2–11.8	9.32 ± 1.15	
PCT (%)	0.17–2.00	0.32 ± 0.31	
PDW (%)	8.5–19.4	13.1 ± 3.10	

**Table 3:** Bivariable and multivariable analysis of predictor variables of anemia among adults with T2DM patients attending Jimma University Medical Center in Jimma, Southwest, Ethiopia 2024.

Variables	Anemia		COR (95% CI)	*p* value	AOR (95% CI)	*p* value
	Yes (%)	No (%)				
Age						
18–25	3 (37.5)	5 (62.5)	Ref		Ref	
26–35	5 (31.2)	11 (68.8)	1.3 (0.22–7.82)	0.760	1.33 (0.22–8.01)	0.757
36–45	8 (14.8)	46 (85.2)	3.45 (0.68–17.37)	0.133	3.42 (0.65–17.83)	0.15
≥ 46	12 (9.8)	110 (90.2)	5.5 (1.17–25.9)	0.031	5.28 (1.07–26.1)	0.041
Sex						
Male	17 (14.7)	99 (85.3)	0.88 (0.39–1.99)	0.75		
Female	11 (13.1)	73 (86.9)	Ref			
Residence						
Urban	16 (14.5)	94 (85.5)	Ref			
Rural	12 (13.3)	78 (86.7)	1.11 (0.49–2.48)	0.81		
Types of medication						
Metformin	12 (11.3)	94 (88.7)	0.62 (0.278–1.39)	0.249		
Metformin + sulfonylurea	16 (17.0)	78 (83.0)	Ref			
FBS level						
< 70 mg/dL	5 (26.3)	14 (73.7)	Ref			
70–125 mg/dL	9 (12.3)	64 (87.7)	2.540 (0.74–8.78)	0.140	2.03 (0.558–7.4)	0.28
≥ 126 mg/dL	14 (13)	94 (87)	2.398 (0.75–7.69)	0.141	1.55 (0.44–5.45)	0.49
Duration of DM						
< 3 years	12 (12.4)	85 (87.6)	Ref			
≥ 3 years	16 (15.5)	87 (84.5)	0.95 (0.42–2.1)	0.895		
BMI						
Normal	16 (13.3)	104 (86.7)	Ref			
Abnormal	12 (15)	68 (85)	0.87 (0.39–1.96)	0.739		

**Table 4:** Logistic regression analysis of leukocytosis and associated variables among adults with T2DM patients attending Jimma University Medical Center in Jimma, Southwest, Ethiopia, 2024.

Variables	Leukocytosis		COR (95% CI)	*p* value	AOR (95% CI)	*p* value
	Yes (%)	No (%)				
Age						
18–25	1 (12.5)	7 (87.5)	Ref		Ref	
26–35	9 (56.2)	7 (43.8)	0.11 (0.011–1.13)	0.063	0.13 (0.013–1.39)	0.092
36–45	6 (11.1)	48 (89.9)	1.14 (0.12–10.96)	0.908	1.15 (0.12–11.33)	0.904
≥ 46	8 (6.6)	114 (93.4)	2.04 (0.22–18.64)	0.529	1.56 (0.16–14.8)	0.698
Sex						
Male	14 (12.1)	102 (87.9)	0.99 (0.42–2.34)	0.97		
Female	10 (11.9)	74 (88.1)	Ref			
Duration of DM diagnosis						
< 3 years	19 (19.6)	78 (80.4)	Ref			
≥ 3 years	5 (4.9)	98 (95.1)	4.77 (1.71–13.36)	0.003	3.1 (1.02–9.5)	0.046
BMI						
Normal	17 (14.2)	103 (85.8)	Ref			
Abnormal	7 (8.8)	73 (91.2)	1.721 (0.679–4.362)	0.25	1.96 (0.7–5.5)	0.2
Residence						
Urban	15 (13.6)	95 (86.4)	Ref	0.43		
Rural	9 (10.0)	82 (90.0)	1.421 (0.591–3.419)			
Types of medication						
Metformin	12 (11.3)	94 (88.7)	0.872 (0.372–2.048)	0.75		
Metformin + sulfonylurea	12 (12.8)	82 (87.2)	Ref			

## Data Availability

The data that support the findings of this study are available from the corresponding author upon reasonable request.
